# Population Genetic Structure of Invasive and Non-Invasive *Streptococcus pneumoniae* Isolates After Fifteen Years of Routine PCV10 Vaccination in Bulgaria

**DOI:** 10.3390/ijms26189028

**Published:** 2025-09-16

**Authors:** Alexandra S. Alexandrova, Vasil S. Boyanov, Kalina Y. Mihova, Preslava M. Hristova, Hristina Y. Hitkova, Yuliya Marteva-Proevska, Raina T. Gergova

**Affiliations:** 1 Department of Medical Microbiology, Medical Faculty, Medical University of Sofia, Zdrave Str. 2, 1431 Sofia, Bulgaria; v.boyanov@medfac.mu-sofia.bg (V.S.B.);; 2 Department of Medical Chemistry and Biochemistry, Molecular Medicine Center, Medical Faculty, Medical University of Sofia, 1431 Sofia, Bulgaria; 3 Department of Microbiology and Virology, Medical University of Pleven, 5800 Pleven, Bulgaria; 4 University Multiprofile Hospital for Active Treatment (UMHAT) Aleksandrovska, Medical University of Sofia, 1431 Sofia, Bulgaria

**Keywords:** *Streptococcus pneumoniae*, vaccine, serotypes, antimicrobial resistance, phylogenetics

## Abstract

*Streptococcus pneumoniae* has been a PCV10 vaccine-preventable agent in Bulgaria since 2010. Our objective is to determine the phylogenetic structure of 170 invasive and non-invasive pneumococcal isolates, focusing on their serotypes and antimicrobial susceptibility. Serotyping was performed using latex agglutination, capsular swelling reaction, and serotype-specific PCRs. Antibiotic susceptibilities were assessed by broth microdilution. MLST was conducted to define the clonal composition. The non-PCV10 serotypes accounted for 88.2%. The predominant invasive pneumococcal disease (IPD) serotypes were 19A (39.3%), 19F (21.4%), 6C (10.7%), 7F (7.1%), and 3 (7.1%). The prevalent NIPD serotypes were 19A (18.3%), 6C (15.5%), 3 (10.6%), 15A (7.7%), and 6A (6.3%). The overall antimicrobial non-susceptibility rates were: benzylpenicillin (55.2%), ceftriaxone (15.2%), cefuroxime (35.8%), amoxicillin-clavulanic acid (38.8%), erythromycin (60.5%), clindamycin (57.0%), tetracycline (43.5%), trimethoprim-sulfamethoxazole (62.9%), and chloramphenicol (13.5%). The multidrug resistance (MDR) strains were 60.5%. The predominant clone CC320, represented 20.0% MDR 19A and 19F strains linked to Taiwan^19F^-14 and GPSC1. CC273/Greece^6B^-22 and CC386 accounted for 5.3% 6A and 6C isolates. Most serotype 3 isolates are associated with CC505, associated with Netherlands^3^-31 and GPSC12. Switching to a conjugate vaccine with broader serotype coverage could reduce the incidence of 19A, 6C, and 15A MDR *S. pneumoniae* clones in our country.

## 1. Introduction

*Streptococcus pneumoniae* (*S. pneumoniae*) is a commensal human pathogen that has garnered the attention of many scientists due to the diseases it can cause, its ability to evade the immune system, and genomic plasticity [1,2].

*S. pneumoniae* can cause both invasive pneumococcal diseases (IPDs) and non-invasive diseases (NIPDs), especially in the extreme age groups—children, and the elderly over 65 years of age due to the immaturity of the immune system in the former group and waning immunity in the latter. The risk factors include immunosuppression, chronic diseases, such as diabetes, sickle cell disease, cardiovascular disease, and renal disease, malnutrition, asplenia, smoking and indoor air pollution, and concomitant viral infection [3,4].

*S. pneumoniae* accounts for NIPDs, like otitis media, non-bacteremic pneumonia, bronchitis, sinusitis, conjunctivitis, and rhinopharyngitis. Nearly every child has experienced NIPD by the age of five years [5,6].

Pneumococcus can cross the blood–brain barrier and may lead to life-threatening IPD infections including pneumonia with bacteremia, septicemia, and meningitis. Less frequent infections caused by *S. pneumoniae* include septic arthritis, osteomyelitis, endocarditis, pericarditis, peritonitis, and soft tissue infections [7,8]. *S. pneumoniae* is a leading global cause of morbidity and mortality. Mortality associated with IPD remains high at 5–35% depending on the site of infection, age, and comorbidity [9]. The mortality rate increases with age. The mortality rate of IPDs in Europe is 15%, and it varies among different age groups, at 4% in children under 15 years of age, 6% in 15–44-year-olds, 11% in 45–64-year-olds, and 21% in those who are over 65 years old [10]. More than 15 years ago, worldwide, IPDs were most prevalent among children, and an estimated 1.6 million deaths occurred annually, mainly due to pneumonia. In 2022, the European Union and European Economic Area reported 17,700 confirmed cases of IPD, with a notification rate of 5.1 cases per 100,000 population, similar to 2018 and 2019. The highest rates were in infants under one year old (13.4 cases per 100,000) and adults aged 65 and older (12.6 cases per 100,000), with males showing higher rates than females across all age groups [https://www.ecdc.europa.eu/en/publications-data/invasive-pneumococcal-disease-annual-epidemiological-report-2022] (accessed on 17 February 2025).

The global burden of pneumococcal diseases has led to the implementation of pneumococcal vaccines in many countries worldwide [11,12,13]. The polysaccharide capsule is the key virulence factor and target for the current pneumococcal vaccines. *S. pneumoniae* can pass through phase variation, allowing it to have different phenotypes of capsules during colonization and invasion, enabling it to evade phagocytosis [14,15,16]. There are at least 100 antigenically distinct polysaccharide capsules or serotypes that differ in their chemical composition, including the arrangement of sugars and glycosidic bonds, and each possesses specific antigenic epitopes [17,18,19]. The composition and coverage of the vaccines against *S. pneumoniae* have changed over time. There are two types of pneumococcal vaccines: conjugate and polysaccharide. Various types of vaccines have been approved for medical use in different countries, leading to updates in immunization programs. The available vaccines from different regions are pneumococcal conjugate vaccines (PCVs) PCV7, PCV10, PCV13, PCV15, PCV20, PCV21, and pneumococcal polysaccharide vaccine PPSV23 [20,21,22]. The first introduced conjugate vaccine was heptavalent vaccine PCV7, which was replaced by subsequent generations [20,23].

The implemented PCVs showed high effectiveness in preventing IPD caused by serotypes in the vaccines. The benefits of PCV mass immunization with PCV10 and PCV13 have dramatically reduced the incidence of IPD, as well as pneumococcal carriage and transmission, and decreased the IPD rates among unvaccinated individuals, including infants too young for the vaccine [24,25,26,27].

PCV10 was the first pneumococcal vaccine introduced in the Bulgarian immunization program in 2010. Immunization is carried out with two vaccines at 2 and 4 months of age and one booster dose at 12 months of age. PCV7 was not used before the introduction of PCV10. The PCV13 and PCV15 vaccines are available in our country, but their administration is not mandatory; they are administered voluntarily. The vaccine included in the National Immunization Program is PCV10, and all the patients or their parents/guardians confirmed that, for the study period, the vaccine administered was PCV10. Since 2023, the Prevenar 13 vaccine has been offered to individuals aged 65 and older as part of the National Program for Improving Vaccination Against Seasonal Influenza and Pneumococcal Infections. The current data shows a very low rate of adults expressing interest in receiving the Prevenar 13 or Prevenar 20 vaccines. The PCV10 immunization in our country was followed by a declining number of IPDs [28], less common vaccinal serotype (VT) pneumococci, and corresponding changes in nasopharyngeal carriage isolates [28,29].

A worrying trend is the high antibiotic resistance of *S. pneumoniae*, which has continued to rise in recent years in Bulgaria and different geographic regions [30,31,32,33].

Since the currently available vaccines provide protection against a limited number of serotypes, their distribution and characteristics warrant careful continuous monitoring. Pneumococci of the same serotype may have genetically distinct backgrounds, different contagiousness abilities, and cause invasive or non-invasive pneumococcal diseases [34,35,36]. Naturally transformable pneumococci may escape vaccine-induced immunity by switching their capsular genes to non-vaccine serotypes [27,31,37,38]. This adaptation has led to serotype replacement, resulting in an increased proportion of pneumococci with non-vaccine serotypes among asymptomatic carriers and symptomatic cases. The pathogen is rapidly adapting to the selective pressure imposed by pneumococcal conjugate vaccines. Some studies discussed that the reduced incidence of pneumococcal meningitis due to vaccination might be only temporary [39]. Multilocus sequence typing (MLST) precisely characterizes pneumococcal isolates into related clonal groups of sequence types (STs) and helps in the investigation of this evolutionary relationship [40].

This research focuses on the epidemiology and genetic adaptation related to vaccine escape in pneumococcal infections. It is crucial to identify the most prevalent pneumococcal serotypes in both IPD and NIPD infections and to understand how serotype prevalence varies across different populations. We examined serotype replacement, the emergence of new serotypes linked to invasive pneumococcal disease, antimicrobial resistance, and the indirect effects of pneumococcal vaccination on trends in antibiotic resistance. All these factors will provide improved insight into pneumococcal infections in our country.

The study aimed to define the current population structure of IPD and NIPD *S. pneumoniae* isolates in our geographic area. We focused on serotype distribution and antimicrobial susceptibility during the recent period from 2020 to 2024, following fifteen years of routine immunization with PCV10 in Bulgaria.

## 2. Results

### 2.1. Studied Population

We analyzed 170 invasive and non-invasive pneumococcal isolates recovered from 100 children (0–16 years of age) and 70 adults (17–82 years of age). The age distribution among children with pneumococcal diseases was as follows: 1 month to 2 years (n = 24), 2 to 7 years (n = 57), and 7 to 16 years (n = 19). Among the adults, 64.3% were aged 17 to 65 years (n = 51), while 35.7% were aged 65 to 82 years (n = 25). Of the children, 98% were PCV10-vaccinated, of which four children received two primary PCV10 doses, and two were without an applied vaccine.

A total of thirty-two *S. pneumoniae* isolates were recovered from specimens associated with systemic infections: cerebrospinal fluids (n = 16), blood (n = 12), and pleural fluids (n = 4) recovered from patients with meningitis, pneumonia plus bacteremia, and pericarditis. The remaining specimens were obtained from pneumococcal strains associated with local infections (n = 138). Some of these specimens included either resident or transient bacterial flora and were collected from various sources: nasopharyngeal samples (n = 45), nasal samples (n = 36), sputum (n = 10), bronchoalveolar lavage (BAL) (n = 5), eye fluids (n = 8), middle ear fluids (n = 29), and wounds/abscesses/localized soft tissue infections (n = 5). Most patients with NIPD had rhinopharyngitis (n = 72), followed by acute otitis media (AOM) (n = 29), bronchitis (n = 16), and non-bacteremic pneumonia (n = 8). Episodes of conjunctivitis were detected in eight cases; five patients exhibited wound infections caused by *S. pneumoniae*. All the IPD isolates (n = 32) were recovered from adult patients (17–82 years of age). The NIPD strains (n = 138) were isolated from various age groups: children up to 2 years of age (n = 24), children aged 2 to 7 years (n = 57), children between 7 and 16 years of age (n = 19), and adults (n = 38). Supplementary Materials Table S1 provides details on the collected samples, their distribution during the investigation period, and the proportions of invasive and non-invasive isolates.

### 2.2. Serotyping

We disclosed twenty-two different serotypes and two non-typeable (NT) strains.

**Figure 1:**
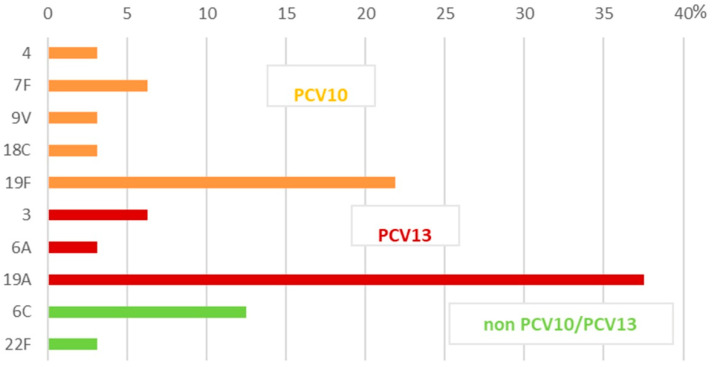
Serotype distribution among 32 invasive *S. pneumoniae* isolates in Bulgaria (2020–2024).

**Figure 2:**
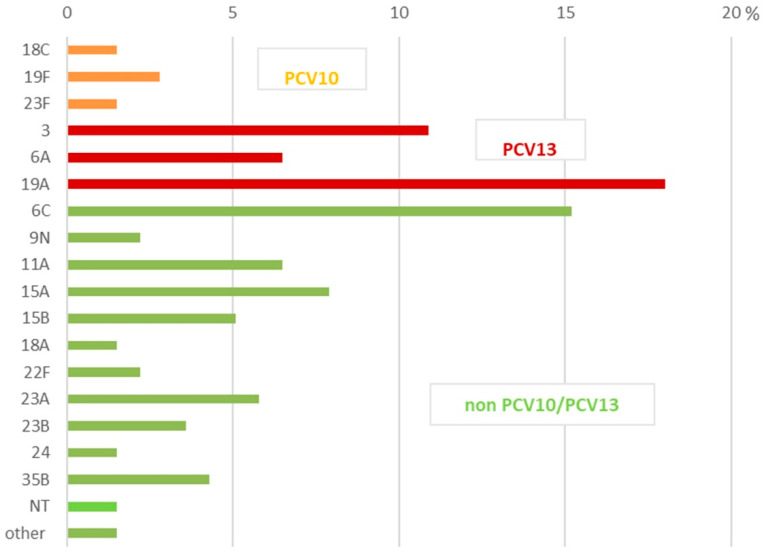
Serotype distribution among 138 non-invasive *S. pneumoniae* isolates in Bulgaria (2020–2024). NT—non-typeable strains with Pneumotest-kit sera; other—strains were positive with one of the pooled sera only: expected to be serotypes/serogroups—25, 38, 43–46, 48 (n = 1), and 31, 40 (n = 1).

The PCV10 serotypes were 11.8%, distributed among 5.8% of the NIPD isolates, and 37.5% in the IPD isolates (Figure 1 and Figure 2). All the other recognized serotypes among the studied population were non-PCV10 serotypes (88.2%). The predominant IPD serotypes were 19A (37.5%), 19F (21.9%), 6C (12.5%), 7F (6.3%), and 3 (6.3%). The IPD isolates were presented mostly by PCV10 serotypes—37.5%. PCV10 serotype 19F and, in particular, serotype 7F emerged among IPD strains, primarily because most patients were adults who had not received the PCV10 vaccine. The remaining recognized serotypes were serotypes 3, 6A, and 19A, which accounted for 46.8% and are not included in PCV10. We named all these serotypes as non-vaccinal for our geographic area. These serotypes are part of the composition of PCV13, PCV15, and PCV20. Non-PCV10/PCV13 serotypes such as 6C and 22F were represented by 15.6% of the isolates.

All the prevalent serotypes among the NIPD isolates were non-vaccinal and mostly from serotypes 19A (18.0%), 6C (15.2%), 3 (10.9%), 15A (7.9%), 6A (6.5%), 11A (6.5%), and 23A (5.8%). The total number of NIPD isolates from non-vaccinal PCV10/PCV13 serotypes was 81 (58.7%). The most prevalent non-PCV10/PCV13 NIPD serotypes were 15A, 11A, and 23A.

Two strains were positive with only one serum and belonged to more than one serogroup or serotype.

**Figure 3:**
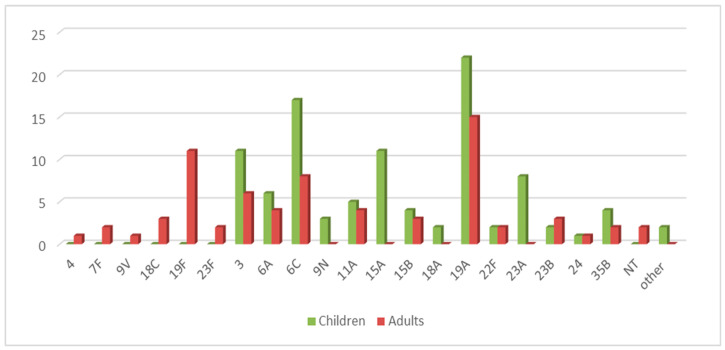
Serotype distribution of invasive and non-invasive *S. pneumoniae* strains among children and adults (2020–2024). Children: 0–16 years of age; adults: 17–82 years of age. NT—non-typeable strains with Pneumotest-kit sera; other—strains were positive with one of the pooled sera only: expected to be serotypes/serogroups- 25, 38, 43–46, 48 (n = 1), and 31, 40 (n = 1).

The most common serotypes among children in descending order were 19A (22.0%), 6C (17.0%), 3 (11.0%), 15A (11.0%), and 23A (8.0%). The serotype distribution according to age is illustrated in Figure 3.

In the group of adult patients above 17 years of age, we found a prevalence of serotypes 19A (21.4%), 19F (15.7%), 6C (11.4%), and 3 (8.6%).

The same leading non-PCV10 serotypes—3, 6A, and 19A—were isolated from both children and adults. The main difference between these two populations is in the highest percentage of 19F (15.7%), which makes the serotypes the second most distributed type in adults in our geographic area.

We observed dominance of serotypes 15A and 23A among children, showing an increase in recent years, with rates of 11.0% and 8.0%.

Among the two non-vaccinated children, we identified serotypes 6C and 15A from the middle ear fluid and nasopharyngeal specimens, respectively.

Table 1 provides information on the available pneumococcal vaccines and the serotypes identified in our study.

Serotypes 6C, 15A, 18A, 23A, 23B, 24, and 35B, identified in our study, are currently not included in any pneumococcal vaccine.

### 2.3. Antimicrobial Susceptibility

The antimicrobial susceptibility testing results indicated that only 22.4% of the strains were susceptible to all the tested antimicrobials, while 77.6% (n = 132) were resistant to one or more classes of antimicrobial agents.

A total of 94 isolates (55.2%) displayed antimicrobial resistance to benzylpenicillin, including both IPD and NIPD strains. Invasive isolates exhibited a resistance rate of 8.8% with low MIC values. Only serotype 19F showed high MIC values exceeding 2 mg/L. Among the NIPD isolates, the resistance to benzylpenicillin reached 46.4%, with the highest MICs observed in serotypes 11A, 15A, 15B, 19A, and 19F. The MIC range of penicillin, ceftriaxone, erythromycin, and clindamycin, with the standard error of the mean for all 170 *S. pneumoniae* isolates, is shown in Supplementary Figure S1. All the ceftriaxone-resistant isolates accounted for 15.2% of the cases, with 3.5% from IPD strains and 11.7% from NIPD. Higher resistance rates were observed for cefuroxime, which reached 35.8% overall. This included 4.1% in the IPD isolates and 31.7% in the NIPD strains. Resistance to amoxicillin-clavulanic acid was recorded at 38.8%, with resistance of 5.3% in patients with bacteremia and sepsis, while the NIPD-resistant strains were 33.5%.

There was significant resistance to macrolides: 60.5% of the studied isolates were non-susceptible to erythromycin, and 57.0% were non-susceptible to clindamycin. The IPD isolates showed equal resistance rates of 9.4% for both erythromycin and clindamycin, while the NIPD isolates demonstrated higher resistance rates of 51.2% for erythromycin and 47.6% for clindamycin. Serotypes 6C, 19A, 19F, and 23A were predominant among the macrolide-resistant strains.

Resistance to tetracycline was also notable, reaching 43.5% overall, with 36.5% in the NIPD isolates and only 7.0% in the IPD strains. The highest resistance rate, at 62.9%, was found for trimethoprim-sulfamethoxazole among the NIPD strains (53.5%), while 9.4% of the IPD isolates were resistant. The lowest resistance was recorded for chloramphenicol at 13.5%, with 2.9% in the IPD strains and 10.6% in the NIPD isolates.

More than half of the strains (60.5%) were multidrug resistant (MDR). The most common MDR combination among all the resistant strains was Pen, Ery, Cli, Tet, and Sxt (47.2%). All the 19A isolates (n = 37) were resistant to benzylpenicillin, macrolides, and tetracycline. Serotypes 19F (n = 8/11; 72.7%), 6C (n = 11/25; 44.0%), 15A (n = 7/11; 63.6%), and 23A (n = 4/8; 50.0%) were frequent in the MDR strains as well. Separate isolates from serotypes 6A, 22F, and 15B also demonstrated MDR profiles.

The isolates were susceptible to the rest of the tested antibiotics: moxifloxacin, levofloxacin, daptomycin, meropenem, ertapenem, linezolid, tigecycline, and vancomycin.

**Figure 4:**
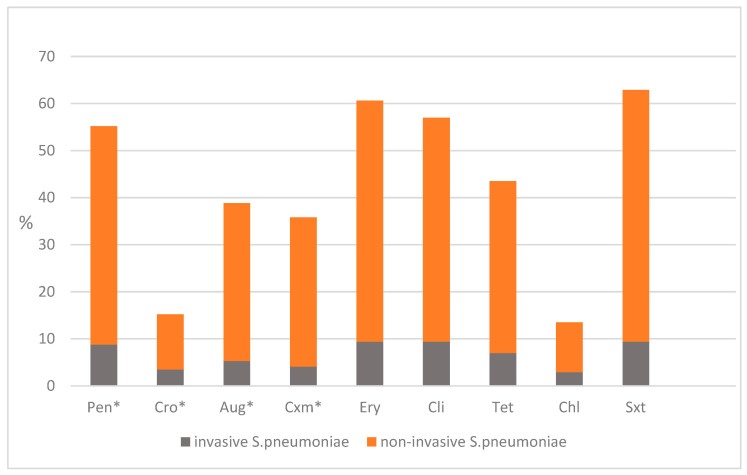
Antimicrobial resistance among 170 invasive and non-invasive *S. pneumoniae* isolates. Pen—penicillin; Cro—ceftriaxone; Aug—amoxicillin–clavulanic acid 2/1; Cxm—cefuroxime; Ery—erythromycin; Cli—clindamycin; Tet—tetracycline; Chl—chloramphenicol; Sxt—trimethoprim-sulfamethoxazole. * The interpretation is based on the EUCAST criteria, 2025. The MIC values for Pen and Cro were determined according to the source of the specimen (IPD—meningitis, pneumonia with bacteremia, and endocarditis; NIPD—all other cases). The MIC values for Aug and Cxm were determined based on the mode of administration—oral or i.v.

The antimicrobial resistance of all the studied invasive and non-invasive isolates is shown in Figure 4.

Figure 4 only illustrates antibiotics that have shown non-susceptibility; all other tested types of Ab that demonstrated full susceptibility are described in the text.

### 2.4. Multilocus Sequence Typing (MLST)

MLST disclosed 19 clonal complexes (CCs) among the examined population of 170 IPD and NIPD *S. pneumoniae* isolates (Table 2). The predominant clone in our country was CC320 (20.0%), composed of 34 MDR isolates from serotypes 19A and 19F, and it was revealed in both the IPD and NIPD isolates. All of the sequence types (STs) from CC320 showed relatedness to reference clone Taiwan^19F^-14 as single-locus variants (SLVs) or double-locus variants (DLVs). Most of these STs were from global pneumococcal sequence cluster (GPSC) 1, the most widely distributed GPS type.

The most prevalent serotype, 19A, was also a part of CC340 (3.5%), CC226 (2.9%), and CC450 (1.8%). All three clusters comprised MDR strains, with relatedness to PMEN clones, Hungary^19A^-6, Denmark^14^-32, and Netherlands^15B^-37. Other major successfully expanded clones encompassed pneumococcal isolates from serotypes 6A and 6C. They were distributed in CC273 (n = 9, 5.3%), which is a PMEN clone Greece^6B^-22, and CC386 (n = 9, 5.3%), where the STs are DLVs of Poland^6B^-20. CC387 and CC2421 grouped only 6C isolates, in which ST387 is an SLV of clone Spain^6B^-2.

Most of the serotype 3 isolates were grouped into CC505 (n = 8), which is associated with Netherlands^3^-31 and GPSC 12, and CC378 (n = 3). The remaining serotype 3 isolates were not closely related to each other but were well-distributed among GPSC types and showed similarities to PMEN clones Sweden^15A^-25 and Greece^21^-30. CC984 comprised MDR 11A isolates from GPSC 18 and was not detected in our country before.

Serotypes 15A and 15B were included in four CCs, either together or separately: CC2613, which consisted of SLVs of Sweden^15A^-25; CC397, which included SLVs or DLVs of Portugal^6A^-41; CC2447, consisting of SLVs of Sweden^15A^-25; and CC275, in which some STs were associated with GPSC48.

The serotype 22F isolates exhibited high antimicrobial resistance and were linked to GPSC19, clustering in CC433. Isolates from serotypes 23A and 23B were represented by CC8029, which encompassed both serotypes, and CC311 and CC272, which covered isolates only from serotypes 23A and 23B, respectively. The isolates in CC311 showed genetic similarity to GPSC7, while those in CC272 were related to the PMEN clone Poland^23F^-16.

Table 3 lists all the clusters of genetically related isolates and the non-clustered isolates identified in the studied population.

The majority of the STs showed relatedness to the GPSC types (65.9%).

**Figure 5:**
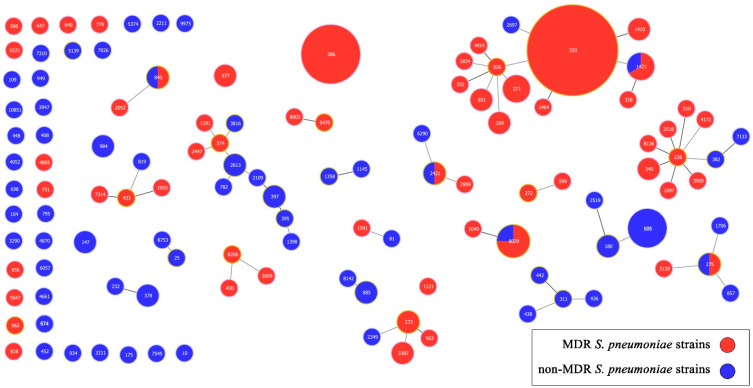
Population snapshot of 170 invasive and non-invasive *S. pneumoniae* isolates based on Phyloviz analysis. Clusters of linked isolates correspond to clonal complexes (CCs). CCs are named after the predominant ST, and, where there is an equal distribution of STs in the CC, it is named after the earlier isolated ST. STs sharing six (single-locus variants—SLVs) and five identical alleles (double-locus variants—DLVs) were assigned to the same CC. Short bold lines connect SLVs; thin long gray lines connect DLVs. STs not assigned to any CC were designed singletons. The size of each circle corresponds to the number of isolates. MDR—multidrug resistance; MDR strain—a strain with resistance to more than three classes of antimicrobials.

The isolates that did not belong to any cluster were classified as singletons (n = 60). For these singletons, we also identified their relatedness to several PMEN clones, including Denmark^12F^-34, Greece^21^-30, Sweden^15A^-25, Tennessee^23F^-4, England^14^-9, CSR^19A^-11, Netherlands^14^-35, Netherlands^8^-33, Spain^23F^-1, Spain^6B^-2, and USA^NT^-43. The population snapshot provided an overview of all 170 sequenced isolates and the clonality observed among them (Figure 5).

## 3. Discussion

Despite being a vaccine-preventable agent, *S. pneumoniae* continues to be one of the leading commensal agents responsible for a significant number of IPD and NIPD cases. The successful dissemination of epidemic clones has been linked to the capsular types, which differ by invasiveness, disease severity, antibiotic resistance profiles, and various virulence factors [34,41,42,43]. Before the introduction of PCV10, our study on 222 invasive *S. pneumoniae* isolates collected from all age groups displayed 59.9% coverage of PCV10 and 78.8% coverage of PCV13. Among the isolates recovered from patients under 5 years of age, the respective coverage rates were 64.2%, 79.1%, and 89.6% [28]. The most prevalent serotypes identified were 6B and 19F, which have since been quickly replaced by emerging serotypes 19A, 6A, and 6C.

In the recent study, the leading serotypes, listed in decreasing order, were 19A, 6C, 3, 15A, and 23A, and the latter two serotypes were distributed only among children.

Serotype 19A was the most common in the IPD and NIPD isolates, distributed in 39.3% and 18.3%, respectively. All the 19A isolates were MDR, and most were clustered in CC320, which is distributed globally and was described in many studies from various geographic areas: Poland, Serbia, Canada, China, and Iran [44,45,46,47]. A study from Ireland showed even the emergence of a sub-clade of 19A clone CC320 associated with vaccine failures [48].

In a comparison with our earlier studies for a pre-vaccine period (2006–2010) for invasive isolates [28], serotype 19A showed a remarkable increase among the same isolates in the current study (*p* = 0.0001 with a significant result at *p* < 0.05).

A global study found that serotype 19A increased before PCV10 or PCV13 introduction and decreaseed at PCV13 sites (age < 5 years: 61–79% decline relative to before any PCV, and in age ≥ 65 years: 7–26% decline) but increased at PCV10 sites, both in children and adults [49,50,51]. Most of the serotype 19A isolates and all the 19F isolates in our study belonged to the same clone, CC320, in which ST320 is an SLV of the MDR international clone Taiwan^19F^-14.

We hypothesize that ST320 originated from the Taiwan^19F^-14/236 lineage, which disseminated in our country in the early 2000s, leading to higher-level β-lactam and macrolide resistance through recombination events that facilitated the emergence of serotype 19A.

Serogroup 6 is also strongly influenced by the capsular switch processes. Serotype 6B was a dominant serotype before the introduction of PCV10 in our country, the third most isolated serotype after 3 and 19F in the pre-vaccine period 2006–2010 [28], but, after the dynamic changes in the serotype distribution, 6B almost disappeared, and there is not one isolate from 6B in the current study. Instead, serotypes 6A and 6C prevailed. During the initial PCV10 mass immunization, 6A was the dominant serotype in our country; however, over time, serotype 6C emerged as the leading serotype from serogroup 6 [29].

A study proved this tendency globally. By 6 years after PCV10 or PCV13 introduction, IPD due to PCV10 serotypes and PCV10-related serotype 6A declined substantially (age < 5 years: 83–99% decline; ≥65 years: 54–96% decline) [49].

The rates of serotype 6C continue to increase in the post-vaccine PCV10 era in our country. In a comparison for 2016–2019, serotype 6C was 11.7% among the NIPD strains, compared to 15.5% for the same types of isolates in the current study [29]. The Fisher exact test statistic value is 0.5007. Although the result is not significant at *p* < 0.05, the values displayed a consistent trend of dominance for serotype 6C.

CC273 and CC386 represented the majority of the 6C and 6A isolates. The ancestor serotype for both CC273 and CC386 was serotype 6B, from international reference clones Greece^6B^-22 and Poland^6B^-20, involving a capsular switching event to serotype 6A and 6C. Serotype 6C comprised a significant number of MDR strains with high MICs for penicillin (>2 mg/L) and high resistance levels for macrolides (>256 mg/L). Other studies also reported high prevalence of 6C and antimicrobial resistance in pneumococcal carriage among children after long-term PCV10 vaccination [52,53].

Serogroup 3 is another major non-PCV10 serotype, predominantly found among AOM isolates in our study. In other studies, serotype 3 was cited as a responsible agent for complicated pneumonia cases [54] or a top-ranked serotype, causing about 9% of cases in children younger than 5 years and 14% in adults aged 50 years or older at both PCV10 and PCV13 sites for IPD [55].

Compared to the antimicrobial resistance of the 19A and 6C isolates, serotype 3 was represented by susceptible or non-susceptible isolates to one or two classes of antimicrobials in our study. The proportion of serogroup 3 in 154 NIPD isolates in the period (2016–2019) was 9.6% [29], with the last four years accounting for 10.6% among the 142 NIPD isolates. The Fisher exact test statistic value is 0.8503, not significant at *p* < 0.05. Nevertheless, this suggests a consistent rate of serogroup 3 in the years following the vaccine introduction.

In addition to the leading serotypes, 3, 6C, and 19A, other serotypes like 15A and 23A are gaining prominence in the population of children. CC2613 comprised only 15A isolates, but other complexes like CC397 and CC275 included both the 15A and 15B isolates, most of them MDR and with genetic relatedness to Portugal^6A^-41, indicating that recombination events may have occurred.

GPSC39 and GPSC7 represented the serotype 23A isolates. In “Pathogen watch”, most of the available genomes from genetic cluster GPSC7 belong to vaccine serotype 23F. Serotype 23F, along with 19F, 14, 9V, and 6B, were responsible for the highest rates of resistance to penicillin and erythromycin worldwide. We suggest that capsular switching and serotype replacement may lead to increases in emerging non-vaccine serotypes that are widespread.

Research from various regions has also highlighted replacement by non-vaccine serotypes. Some investigations demonstrated that vaccine types have almost disappeared in children [31,33,56].

A study conducted in Lebanon found that a serotyping analysis revealed 31.3% of the isolates corresponded to vaccine serotypes covered by PCV13 and 50% to those in PCV20. The most commonly identified serotypes were 11A, 19F, and 23A, with serogroup 24 accounting for 37.5% of the serotyped isolates [57]. Our results also demonstrated a rise in 11A and 23A cases.

In Croatia, the prevalence of carriage among healthy children increased from 19.9% to 28.7%, mainly due to a rise in non-vaccine serotypes like 6C, 11A, 19A, and 23A, which were significantly higher in healthy children who were exposed. These serotypes were also more prevalent in children with pneumonia or acute otitis media [54].

Investigations from Spain where mass immunization occurs with PCV13 exhibited that the most common serotypes in decreasing order were 3, 19A, 8, 7F, 1, 6C, 11A, 22F, and 14, and the vaccine types (PCV13 + 6C) were responsible for around 50% of the IPD episodes among adults, with a significant decrease in serotypes 6C and 7F [37].

In the UK, the serotype distribution of carriage in children revealed serotypes 11A, 23B, 15B, 21, 10A, and 15A, and, for IPD in children, the most common serotypes were 12F, 24F, 23B, 15B/C, 10A, and 22F [39]. Similarly to our findings, the new emerging serotypes, which were not even detected in the pre-vaccine era, were from serogroup 15 and serogroup 23 [28].

The results of our study demonstrate that we cannot definitively link a specific serotype to either invasive or non-invasive diseases as various serotypes have been identified in both young children and adults with differing conditions, such as meningitis, otitis media, or rhinopharyngitis.

Regarding the relationship between serotypes and multidrug resistance, we noticed that certain serotypes, including 19A, 19F, 6C, and 15A, are associated with MDR. In contrast, serotype 3 is predominantly found in susceptible strains. Various socioeconomic and demographic factors may influence serotype patterns and antibiotic resistance [58]. The age distribution is of high importance because *S. pneumoniae* tends to attack the extreme age groups. Children under <5 years of age are the primary carriers and transmitters of *S. pneumoniae*, and the elderly are at high risk for IPD due to waning immunity [3,59]. The vaccines for these age groups can effectively reduce the incidence of pneumococcal diseases. Immunization in childhood can indirectly lower the occurrence of IPD in adults through the development of herd immunity [60]. Socioeconomic factors, such as urbanization and school and kindergarten attendance, can increase transmission and carriage. Carriers may spread highly resistant clones of *S. pneumoniae* [61,62]. Higher inappropriate antibiotic use also leads to higher selective pressure for resistant strains and promotes multidrug resistance [63].

Differences in vaccine strategies across various geographic regions may also lead to resistance in prevalent serotypes, which can rapidly spread due to travel and migration between countries [64].

The public health strategy for pneumococcal diseases should take into account epidemiological and social factors, rapid recombination events, local serotype distributions, and resistance data. Continuous surveillance is essential to monitor the impact of vaccines, detect serotype replacement, and update vaccine formulations [65,66,67].

Limitation statement: The study was conducted during a period that largely coincided with the COVID-19 pandemic, resulting in a relatively small number of strains collected, especially for invasive isolates. Despite this, the collection of *S. pneumoniae* isolates provided a thorough understanding of the current situation regarding the emerging serotypes, clonal structures, and genetic lineages that exhibit multidrug resistance.

Please note that percentages presented without totals do not indicate absolute numbers, and the percentage of non-vaccine-covered strains is expected to rise due to the decline in vaccine-covered strains.

Multilocus sequence typing (MLST) is based on seven housekeeping genes, and the method may not capture the complete genetic diversity present in the genome. In some cases, the same sequence type (ST) has been found in more than one global pneumococcal sequence cluster (GPSC). This can occur due to the highly recombinogenic nature of pneumococcal genomes, where strains that are not closely related may acquire similar allelic profiles or where different lineages might independently evolve similar housekeeping gene sequences, resulting in the same ST.

## 4. Materials and Methods

### 4.1. Patients and Specimen Collection

The patient population included all individuals diagnosed with either IPD or NIPD, from which *S. pneumoniae* was recovered from sterile or non-sterile areas of the human body, respectively. A patient is considered to have a pneumococcal infection if they exhibit clinical signs of a disease typically caused by *S. pneumoniae*, supported by laboratory confirmation through culture isolation, PCR identification, or antigen detection for serotyping. In the period 2020–2024, we collected 170 IPD and NIPD *S. pneumoniae* isolates, recovered from patients of different ages. Three major microbiological laboratories participated in the project: the Department of Medical Microbiology at the Medical University of Sofia, which provided 37 strains distributed across the following age groups: 0–7 years (n = 16), 8–16 years (n = 8), and adults aged 17–82 years (n = 13). The microbiological laboratory from University Multiprofile Hospital for Active Treatment (UMHAT) “Aleksandrovska” in Sofia participated with 52 strains recovered from patients 0–7 years (n = 37), 8–16 years (n = 6), and adults between 17 and 82 years (n = 9), and the UMHAT “Georgi Stranski” in Pleven contributed with 28 isolates from children up to 7 years, 5 children between 8 and 16 years, and 48 adults. Clinical and demographic data (age, diagnosis, and vaccine status) were collected.

NIPD was defined as pneumococcal infection detected in the nasopharynx, eye, ear, sputum, or nose/throat specimens, and in which no sterile site isolates were collected from the same patient.

The IPD isolates were found at normally sterile sites such as blood and cerebrospinal fluid, leading to IPD, including septicemia, meningitis, and pneumonia with bacteremia.

In terms of age, the patients were categorized into two groups: children from 2 months to 15 years of age, and adults, between 16 and 82 years of age. Pneumococcal immunization started 15 years ago in our country (2010), and, currently, all newborns are vaccinated with PCV10. The vaccination against pneumococcal infections in Bulgaria is carried out with two vaccines at 2 and 4 months of age and re-immunization with one vaccine at 12 months of age. Children who had received 2 primary doses +1 booster dose of PCV10 were defined as PCV10-vaccinated.

### 4.2. Identification

The strains were identified with conventional microbiological tests, such as optochin test and bile solubility test, and were confirmed by amplification of the autolysin-encoding gene, *lytA*, which enables reliable diagnosis [68].

### 4.3. Serotyping

Serogrouping and serotyping of the *S. pneumoniae* isolates were performed by latex agglutination method, capsular swelling reaction using commercial serotype-specific factor antisera (SSI, Copenhagen, Denmark), and PCR-serotyping for serogroup 6, as described previously [69,70]. The Neufeld test is performed with group antisera that react with all serotypes in the group. After growing a pure *S. pneumoniae* isolate in a Todd–Hewitt broth (HiMedia Company, Mumbai, Maharashtra, India) for 24h at 36 °C, a drop of the culture was mixed with the group antisera and was inspected in a phase contrast microscope. The pneumococcal capsule becomes visible and swollen, and the pneumococci agglutinate if the reaction is positive as a result of in situ immunoprecipitation between the pneumococcal capsular polysaccharide and its homologous antibodies in the antiserum. The ultimate identification of the serotypes in the group is carried out by serotyping with factor antisera. A pure bacterial culture was mixed with the solutions consisting of latex particles coated with specific antiserum. If the test is positive, agglutination is revealed within 10 s, resulting in large visible aggregates. We conducted PCR serotyping to simultaneously detect serotypes 6A and 6C, which share high similarity in their cps loci. The presence of serotypes 6A and 6C was confirmed by amplifying a 149 bp product from the *wciP* gene. To specifically identify serotype 6C, we targeted the *wciNβ* gene, resulting in a 359 bp product. For isolates of serotype 6B, we performed PCR using primers designed to amplify a segment of the *wciP* gene, which produced a 155 bp product. The primers used in our study were previously published by Jin et al. The non-typeable (NT) strains were tested by latex agglutination and capsular swelling reaction.

### 4.4. Antimicrobial Susceptibility Testing

The minimum inhibitory concentrations (MICs) were determined by the broth microdilution method using the Sensitre custom plate format (TREK diagnostic systems, Thermo Fisher Scientific Inc., Waltham, MA, USA), plate code: STP6F, and according to the EUCAST breakpoints for IPD and NIPD isolates, v.15.0, 2025 [71]. An inoculum of 0.5 McFarland Standard was prepared by suspending 3–5 colonies of a pure culture of the tested strain in water using a nephelometer. A Mueller-Hinton Broth with lysed horse blood (Sensititre Mueller Hinton Broth, Thermo Fisher Scientific Inc., Waltham, MA, USA) was used for the susceptibility testing. A bacterial suspension of 100 μL was mixed into the broth, and 100 μL of this mixture was inoculated into each well of the plate. The Sensititre plate was then sealed and incubated at 36 °C in a non-CO_2_ incubator for 24 h. After incubation, the results were manually read using the Sensititre Manual Viewbox (Thermo Fisher Scientific Inc., Waltham, MA, USA)

The M.I.C.Evaluator™ (M.I.C.E.™) strips (Oxoid, Basingstoke, Hampshire, UK) were used to evaluate the minimum inhibitory concentrations (MICs) of penicillin, ceftriaxone, erythromycin, and clindamycin according to manufacturer’s instructions. The standard quality-control strain was *S. pneumoniae* ATCC 49619. MDR was defined by resistance to at least three or more classes of antimicrobial agents.

### 4.5. Multilocus Sequence Typing (MLST)

MLST was carried out as described by Enright [72]. Seven housekeeping genes were sequenced and compared to the pneumococcal MLST database http://pubmlst.org/spneumoniae (accessed on 10 January 2025) to identify the alleles and sequence types (STs). Clusters of related sequence types (STs) were grouped into clonal complexes (CCs) by use of the goeBURST algorithm of PHYLOViZ http://phyloviz.net/ (accessed on 15 January 2025). GPSC types of STs were determined using the Global Pneumococcal Sequencing Project: GPS https://www.pneumogen.net (accessed on 20 January 2025), which represents the international genomic definition of the pneumococcal lineages [73]. The Pathogen Watch platform was utilized for comparative genomic epidemiology and surveillance https://pathogen.watch/genomes/all?genusId=1301&speciesId=1313 (accessed on 21 January 2025).

### 4.6. Statistical Analysis

Statistical analyses were conducted using IBM SPSS Statistics for Windows version 19.0 (IBM Corp., New York, NY, USA). The threshold for statistical significance was set at a *p*-value of ≤0.05.

## 5. Conclusions

Serotype replacement is a consequence of new recombinant antigenic variants, which escape the PCV10. We determined a significant rate of incidence of non-vaccine PCV10 and PCV13 serotypes. The spread of antimicrobial-resistant clones resulted in a high prevalence of MDR strains. More than half of the studied isolates were MDR, particularly distinctive for serotypes 19A, 6C, and 15A. Future vaccine development must adapt to local serotype patterns. Continuous serotype and resistance monitoring is essential for future vaccination and public health.

## Figures and Tables

**Table 1:** Serotypes covered by pneumococcal vaccines.

Vaccine	Serotypes
*PCV10	**1, 4, 5, 6B, 7F, 9V, 14, 18C, 19F, 23F**
**PCV13**	**1, 3, 4, 5, 6A, 6B, 7F, 9V, 14, 18C, 19A, 19F, 23F**
**PCV15**	**1, 3, 4, 5, 6A, 6B, 7F, 9V, 14, 18C, 19A, 19F, 22F, 23F, 33F**
**PCV20**	**1, 3** **, 4** **, 5, 6A** **, 6B, 7F** **, 8, 9V** **, 10A, 11A** **, 12F, 14, 15B** **, 18C** **, 19A** **, 19F** **, 22F** **, 23F** **, 33F**
**PCV24**	**1, 2, 3** **, 4** **, 5, 6A** **, 6B, 7F** **, 8, 9N** **, 9V, ** **10A, 11A** **, 12F, 14, 15B** **, 17F, 18C** **, 19A** **, 19F** **, 20, 22F** **, 23F** **, 33F**
**PPV23	**1, 2, 3** **, 4** **, 5, 6B, 7F** **, 8, 9N** **, 9V** **, 10A, 11A** **, 12F, 14, 15B** **, 17F, 18C** **, 19A** **, 19F** **, 20, 22F** **, 23F** **, 33F**

*PCV—pneumococcal conjugate vaccine; **PPV—pneumococcal polysaccharide vaccine. The serotypes detected in our study are marked in red.

**Table 2:** Distribution of clinical specimens and serotypes with their connection to vaccines.

IPD/NIPD ^1^ Isolates	Patient Specimens ^2^	n ^3^ (%) of All Isolates	Serotype (n of Isolates)	*PCV10, PCV13, non-PCV10/PCV13 Serotypes
**Invasive isolates** **n = 32**	CSF	16 (9.4)	4 (1), 7F (2), 19F (4)	PCV10
			3 (2), 19A (7)	PCV13
	Blood	12 (7.1)	9V (1), 19F (2)	PCV10
			6A (1), 19A (4)	non-PCV10
			6C (3)	PCV13
			22F (1)	non-PCV10/PCV13
	Pleural fluids	4 (2.3)	18C (1), 19F (2)	PCV10
			19A (1)	PCV13
**Non-invasive isolates** **n = 138**	Nph samples	45 (26.5)	23F (1)	PCV10
			3 (2), 6A (4), 19A (8)	PCV13
			6C (4), 11A (6), 15A (8), 15B (4), 22F (2), 23A(1), 23B (3), 35B (2)	non-PCV10/PCV13
	Nose	36 (21.2)	19F(1)	PCV10
			3 (2), 6C (6), 19A (8)	PCV13
			9N (1), 11A (3), 15A (3), 23A (5), 24 (2), 35B (4), Other (1)	non-PCV10/PCV13
	Sputum	10 (5.8)	18C (1), 19F(2)	PCV10
			6A(1)	non-PCV10
			6C (1), 9N (1), 15B (1), 22F (1), 23B (2)	non-PCV10/PCV13
	BAL	5 (2.9)	23F (1)	PCV10
			3 (2), 19A (1)	PCV13
			NT (1)	non-PCV10/PCV13
	Eye fluids	8 (4.7)	18C (1)	PCV10
			6C (1), 19A (2)	PCV13
			15B (2), NT (1), Other (1)	non-PCV10/PCV13
	Middle ear fluid	29 (17.2)	3 (9), 6A (3), 19A (5)	PCV13
			6C (8), 15C (2), 18A (2)	non-PCV10/PCV13
	Wound	5 (2.9)	6A (2), 19A (2)	non-PCV10
			9N (1)	non-PCV10

^1^ IPD—invasive pneumococcal disease; NIPD—non-invasive pneumococcal disease. ^2^ Patient specimens abbreviations: CSF—cerebrospinal fluid; Nph—nasopharynx; BAL—bronchoalveolar lavage; ^3^ n—number of strains; *PCV—pneumococcal conjugate vaccine.

**Table 3:** Phylogenetic characteristics of 170 invasive and non-invasive *S. pneumoniae* strains.

	Serotype	CC ^1^	ST ^2^ (n)	GPSC ^3^ Type (ST)	PMEN ^4^
**PCV10 serotypes**	4	-	7026 (1)	**70** (7026)	**DLV of Denmark^12F^-34**
	7F	-	6057 (1), 5374 (1)	**32** (6057)	-
	9V	-	4952 (1)	-	-
	18C	-	843 (1), 1358 (1), 9975 (1)	**22** (843) **11** (1358, 9975)	**SLV of Greece^21^-30** (ST1358)
	23F	-	647 (1), 408 (1)	**13** (647) **3** (408)	-
	19F	**CC320**	320 (2), 328 (1), 1421 (2), 1464 (1), 2697 (1), 4414 (1), 651 (1), 926 (1), 1834 (1)	**1** (320, 1421, 1464, 2697, 4414, 651, 926)	**SLV of Taiwan^19F^-14** (STs 651, 926, 1834) **DLV of Taiwan^19F^-14** (STs 320, 328, 1421, 1464, 2697)
**non-PCV10 serotypes**	19A		320 (13), 271 (3), 299 (2), 1410 (2),352 (1), 651 (1), 1421 (1)	**1** (271,320, 651, 1421)	**SLV of Taiwan^19F^-14** (299,352,651) **DLV of Taiwan^19F^-14** (320, 271, 1410, 1421)
	3	**CC505**	505 (5), 180 (2), 2519 (1)	**12** (505, 180)	**Netherlands^3^-31** (180) **SLV of Netherlands^3^-31** (2519) **DLV of Netherlands^3^-31** (505)
	3	**CC378**	378 (2), 232 (1)	**83** (378, 232)	-
	3	-	782 (1), 1145 (1), 4661(1), 3290 (1), 4805 (1), 7545 (1)	**9** (782) **11** (1145) **43** (4661) **36** (3290) **51** (7545)	**SLV of Sweden^15A^-25** (782) **DLV of Greece^21^-30** (1145)
	6A, 6C	**CC273**	273 (2), 147 (3), 885 (2), 2349 (1), 8142 (1)	**23** (273, 147)	**Greece^6B^-22** (273) **SLV of Greece^6B^-22** (2349) **DLV of Greece^6B^-22** (147, 885, 8142)
	6A	-	5470 (1), 81 (1)	**13** (5470) **16** (81)	-
	6A, 6C	**CC386**	386 (9)	**47**	**DLV of Poland^6B^-20** (386)
	6C	**CC3387**	3387 (2), 104 (1)	**23** (3387, 104)	**SLV of Spain^6B^-2** (3387, 104)
	6C	**CC2421**	2421 (2), 6290 (1), 2909 (1)	**37** (2421, 6290, 2909)	-
	6C	**-**	640 (1), 7310 (1), 8003 (1), 5647 (1), 751 (1), 3211 (1), 674 (1), 818 (1),1121 (1)	**23** (640, 1121) **13** (7310, 8003) **52** (5647) **37** (751, 3211)	**DLV of Tennessee^23F^-4** (674)
	9N	-	4870 (1), 843 (1), 2211 (1)	**17** (4870) **22** (843) **26** (2211)	-
	11A	-	25 (1), 175 (1), 596(1), 630 (1), 934(1), 8753 (1), 596(1), 934 (1)	**18** (25, 8753) **55** (175)	**SLV of England^14^-9** (25) **CSR^19A^-11** (175)
	11A	**CC984**	984 (2), 6221 (1)	**18** (984, 6221)	-
	15A	**CC2613**	2613 (2),374 (1), 1191 (1)	**9** (2613, 374, 1191)	**SLV of Sweden^15A^-25** (2613, 374, 1191)
	15A		5139 (1), 2052 (1), 962 (1), 10 (1)	**11** (5139) **22** (2052)	-
	15A,15B	**CC397**	397 (2), 395 (1)	**29** (395)	**SLV of Portugal^6A^-41** (395) **DLV of Portugal^6A^-41** (397)
	15A, 15B	**CC275**	275 (2), 3139 (1), 857 (1), 1706 (1)	**48** (275)	-
	15B	**CC2447**	2447 (1), 3816 (1)	**9** (2447,3816)	**SLV of Sweden^15A^-25** (2447, 3816)
	18A	-	795 (1), 2109 (1)	-	-
	19A	**CC450**	450 (1), 2609 (1), 8206 (1)	**4** (450)	**SLV of Netherlands^15B^-37** (2609, 8206) **DLV of Netherlands^15B^-37** (450)
	19A	**CC340**	340 (2), 7133 (1), 4172 (1), 8136 (1), 2518 (1)	-	**SLV of Hungary^19A^-6** (340, 7133) **DLV of Hungary^19A^-6** (4172, 8136, 2518)
	19A	**CC226**	226 (1), 382 (1), 510(1), 1897 (1), 3909 (1)	**738** (226)	**SLV of Denmark^14^-32** (226, 382) **DLV of Denmark^14^-32** (510, 1897, 3909)
	22F	**CC433**	433(1), 1955 (1), 7314 (1)	**19** (433, 1955,7314)	-
	22F	-	698 (1)	**61** (698)	-
	23A, 23B	**CC8029**	8029 (4),1040 (1)	-	-
	23A	-	442 (1), 656 (1), 577 (1)	**39** (656)	**SLV of Netherlands^14^-35** (656, 577)
	23A	**CC311**	311(1), 438 (1)	**7** (311, 438)	
	23B	**CC272**	272 (1), 599 (1)		**SLV of Poland^23F^-16** (272)
	23B	-	1398 (1)	**29** (1398)	-
	24	-	109(1), 819 (1)	-	-
	35B	-	452 (1), 577 (1), 779 (1), 963 (1), 1591(1), 3947 (1)	**75** (452) **715** (779) **16** (1591)	**SLV of Netherlands^8^-33** (577) **SLV of Spain^23F^-1** (1591) **DLV of Spain^6B^-2** (963)
	NT ^5^	-	436(1), 448 (1)	**7** (436) **60** (448)	**USA^NT^-43** (448)
	Other ^6^	-	949 (1), 10851 (1)	**304** (10851)	**DLV of Netherlands^14^-35** (949)

^1^ CC—clonal complex; ^2^ ST—sequence type; ^3^ GPSC—global pneumococcal sequence cluster; ^4^ PMEN—Pneumococcal Molecular Epidemiology Network (SLV—single-locus variant; DLV—double-locus variant); ^5^ NT—non-typeable strains with Pneumotest-kit sera; ^6^ other—trains were positive with one of the pooled sera only: expected to be serotypes/serogroups- (n = 1, 25, 38, 43–46, 48), and 31, 40 (n = 1).

## Data Availability

All datasets generated or analyzed during the study are included in the manuscript.

## Supplementary Materials

The following supporting information can be downloaded at: https://www.mdpi.com/article/10.3390/ijms26189028/s1.
