# Supplementary material for: Population Genetic Structure of Invasive and Non-Invasive Streptococcus pneumoniae Isolates After Fifteen Years of Routine PCV10 Vaccination in Bulgaria
Source: Int J Mol Sci. 2025 Sep 16;26(18):9028. doi: 10.3390/ijms26189028 (PMC12469886; doi:10.3390/ijms26189028)
Supplement: Supplementary file 1 [file ijms-26-09028-s001.zip › ijms-3797068 Table S1.pdf]

Table S1. Distribution of collected samples during the investigation period and associated diseases.

Patients		n (%)	IPD isolates (n)		Year of isolation (n)					NIPD isolates (n)		Year of isolation (n)				
					2020	2021	2022	2023	2024			2020	2021	2022	2023	2024
**Children 0-16 y n=100**	Children 0-2	24 (14.1)	-		-	-	-	-	-	24 (14.1)	Rhinopharyngitis (13)	5	4	-	2	2
											AOM (6)	1	-	-	1	4
											Bronchitis (3)	-	-	3	-	-
											Conjunctivitis (2)	-	-	-	2	-
	Children 2-7	57 (33.5)	-		-	-	-	-	-	57 (33.5)	Rhinopharyngitis (30)	4	3	8	6	9
											AOM (13)	7	-	-	1	5
											Bronchitis (6)	2	-	2	1	1
											Pneumonia (4)	2	1	-	-	1
											Conjunctivitis (4)	-	-	-	3	1
	Children 7-16	19 (11.2)	-		-	-	-	-	-	19 (11.2)	Rhinopharyngitis (5)	1	1	-	-	3
											AOM (7)	1	-	-	2	4
											Bronchitis (3)	2	-	1	-	-
											Pneumonia (2)	-	-	-	1	1
											Conjunctivitis (2)	-	2	-	-	-
**Adults 17-82y n=70**	Adults 17- 65	45 (26.5)	11 (34.3)	Pneumonia plus bacteremia (7)	4	-	2	-	1	-	Rhinopharyngitis (24)	6	4	8	3	3
				Meningitis (4)	-	1	2	1	-	-	Bronchitis (3)	-	-	1	-	2
											AOM (3)	-	1	-	1	1
											Wound cases (4)	2	1	1	-	-
	Adults 65 - 82	25 (14.7)	21 (65.7)	Meningitis (12)	3	4	3	1	1	-	Bronchitis (1)	-	-	-	-	1
				Pneumonia plus bacteremia (5)	1	3	-	-	1	-	Pneumonia (2)	-	2	-	-	-
				Pericarditis (4)	2	2	-	-	-	-	Wound cases (1)	1	-	-	-	-
**Total**	**n=170**	**100%**	**n=32 (18.8)**		**10**	**10**	**7**	**2**	**3**	**n=138 (81.2)**		**34**	**19**	**24**	**23**	**38**
