# Supplementary material for: Population Genetic Structure of Invasive and Non-Invasive Streptococcus pneumoniae Isolates After Fifteen Years of Routine PCV10 Vaccination in Bulgaria
Source: Int J Mol Sci. 2025 Sep 16;26(18):9028. doi: 10.3390/ijms26189028 (PMC12469886; doi:10.3390/ijms26189028)
Supplement: Supplementary file 1 [file ijms-26-09028-s001.zip › ijms-3797068 Figure S1.pdf]

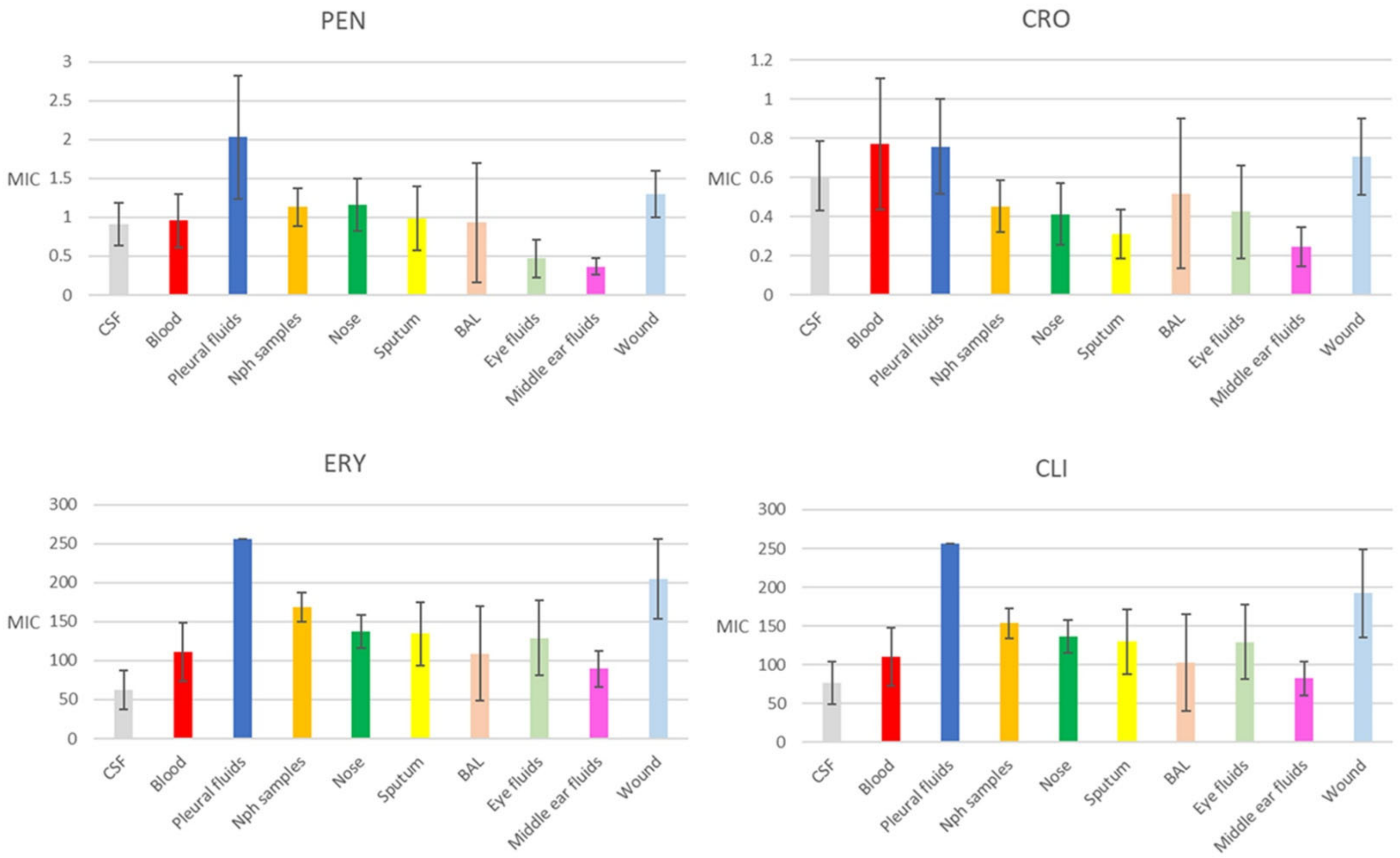

Figure S1. MIC range of antimicrobial agents with standard error of the mean for 170 *S. pneumoniae* isolates.

Notes: MIC – Minimal inhibitory concentration. Pen – penicillin, Cro - ceftriaxone, Ery – erythromycin, Cli – clindamycin. CSF – cerebrospinal fluid, Nph samples- nasopharyngeal samples, BAL – bronchoalveolar lavage.

*The interpretation is based on the EUCAST criteria, 2025. The MIC values for Pen and Cro are determined according to the source of the specimen (IPD – meningitis, pneumonia with bacteremia, and endocarditis; NIPD – all other cases).
